# Hybrid computational intelligence framework for accurate wind power forecasting and grid integration applications

**DOI:** 10.1038/s41598-026-66248-z

**Published:** 2026-08-11

**Authors:** Tariq Alkhrissat, Firas Badri Abed, Saeed Aldulaimi, Jasgurpreet Singh Chohan, M. K. Ranganathaswamy, Premananda Pradhan, Zokir Mamadiyarov, Hayitov Abdulla Nurmatovich

**Affiliations:** 1 Faculty of Engineering, Hourani Center for Applied Scientific Research, Al- Ahliyya Amman University, Amman, Jordan; 2 Department of Mechanical Engineering, Al-Turath University College of Engineering, Baghdad, 10013 Iraq; 3 College of Administrative Sceiences, Applied Science University, Sitrah, Kingdom of Bahrain; 4 Marwadi University Research Center, Department of Mechanical Engineering, Faculty of Engineering & Technology, Marwadi University, Rajkot, Gujarat India; 5 Faculty of Engineering, Sohar University, PO Box 44, Sohar, PCI 311 Oman; 6 Department of Mechanical Engineering, School of Engineering and Technology, JAIN (Deemed to be University), Bangalore, Karnataka India; 7 Department of Mechanical Engineering, Siksha ’O’ Anusandhan (Deemed to be University), Bhubaneswar, 751030 Odisha India; 8 Mamun University, Khiva, Uzbekistan; 9 Faculty of Technology, Urgench State University, Urgench City, Uzbekistan; 10 Department of Financial Engineering, Termez University of Economics and Service, Termez, Uzbekistan

**Keywords:** Wind power forecasting, Hybrid machine learning, Computational intelligence, Renewable energy integration, Power grid engineering, Nature-inspired optimization, Energy science and technology, Engineering, Mathematics and computing

## Abstract

Accurate wind power forecasting is essential for the reliable operation and large-scale integration of renewable energy into modern power grids. This study develops and systematically evaluates a hybrid computational intelligence framework that integrates advanced machine learning models with nature-inspired optimization algorithms for wind power prediction. CatBoost (CAT), Long Short-Term Memory (LSTM), and Adaptive Neuro-Fuzzy Inference System (ANFIS) models were optimized using Cuckoo Search Optimization (CSO) and the Stochastic Paint Optimizer (SPO) to determine the most effective model–optimizer configuration under variable wind conditions. A comparative analysis demonstrates that the CAT–SPO hybrid model achieved the best predictive performance, yielding a test RMSE of 0.0338 and an R² of 0.984, outperforming alternative configurations. Feature relevance analysis and multicollinearity assessment using the Variance Inflation Factor (VIF) identified hub-height wind speed (100 m) as the dominant predictor (32.5% relative importance; VIF ≈ 3.96), while lower-height wind speed (10 m) was excluded due to high collinearity. Wind gust measurements at 10 m retained substantial explanatory contribution (≈ 19.2% importance; VIF ≈ 4.34), highlighting the role of short-term atmospheric variability in power modeling. The proposed framework enhances forecasting reliability and supports improved grid stability, reserve allocation, renewable energy integration, and data-driven operational planning. These findings advance intelligent energy management systems and sustainable power grid engineering.

## Introduction

Accurate wind power forecasting plays a critical role in ensuring the stability, reliability, and economic efficiency of modern power systems with high penetration of renewable energy^1^. Due to the inherently intermittent and nonlinear nature of wind, predicting power output remains a challenging task, particularly for short-term operational planning and grid integration^2,3^. Even small forecasting errors can lead to inefficient reserve allocation, increased operational costs, and instability in electricity markets^4–6^. Traditional forecasting methods very often fail to handle the complexity that arises with increasing amounts of wind data. Hence, one of the main demands in scientific research on renewable energy has become the development of high-precision algorithms for the estimation of the prediction values^7^. Data-driven methods that heavily rely on historical data and real-world correlations are more efficient than physical models based on complex fluid-dynamic simulations and numerical weather prediction. The use of artificial intelligence (AI) and machine learning has made flexible systems that are capable of finding nonlinear relationships and can also change with the wind pattern^8,9^. Hybrid methods that integrate multiple forecasting paradigms improve performance by leveraging the strengths of each strategy.

Various papers examine wind energy from both policy and engineering perspectives. Several of the recent contributions not only explain different but also interrelated elements of this environment. Thus, the paper “The role of renewable energy in achieving sustainable development goals” by Victoria Agbakwuru, Peter Ofuje Obidi, and Ojonimi Segun Salihu aims to showcase the entire process in which renewable energy progressively meets the UN Sustainable Development Goal requirements, emphasizing the social, economic, and environmental co-benefits of the renewables, besides proposing ways to remove the obstacles to their adoption. The authors claim the novelty of the work lies in the bottom-up approach to practical adoption routes, harmonized with policy tool strategies that put renewable energy in the spotlight as the main lever of development rather than just another technology choice^10^. Further determined to model resource variability for operations, Wind Power Scenario Generation Considering Wind Power Variations by Longpeng Ma, Chen Wu, and Kaihui Nan addresses the methodological challenge of wind uncertainty representation. The authors propose a scenario-generation approach that concisely reflects the actual temporal changes in wind power and provides operationally valid outlines for reserve sizing and dispatch. The contribution is a step forward in stochastic intermittency representation for system planning^11^. In relation to the device and system level, Comprehensive overview of wind turbines by Wadirin Wadirin, Dendy Adanta, and Dewi Puspita Sari attempts to cover turbine evolution from the historical and technical points of view, development of rotors, drivetrains, and control systems, and resistance capacity and economic impacts due to these advancements are the author’s main line of argument here; the article’s practical novelty is seen in the bringing together of hardware developments that lead to the forecast of hub-height and the demand for more accurate operational models^12^.

Complementing technology surveys, Advancing Sustainable Power by Murad Ali Khan presents a cross-technology assessment, comparing solar, wind, biomass, and hydro, that identifies the trends of efficiency, costs, and environmental trade-offs; the consolidation of Khan is unique in linking the techno-economic trajectories of different technologies with the planning of the strategy for their deployment and integration, which relies on correct forecasting and systems engineering^13^. In the paper “Harnessing the Power of Renewable Energy” by Shivani Pawar, the author scrutinizes how the technology is performing and its viability from an economic point of view, with a focus on the efficiency gains and cost reductions in wind and solar; additionally, the paper provides a link between the learning-curve data to emissions reduction potential and stating that the improved prediction of renewable energy will make their operational value greater by minimizing the uncertainty premiums in the markets and operations^14^.

Recent advancements in metaheuristic optimization have introduced novel algorithms inspired by physical and mathematical principles. For instance, the Schrödinger Optimizer is a quantum-inspired metaheuristic based on wave–particle duality, employing a dual update mechanism that balances exploration and exploitation in complex search spaces^15^. Similarly, the Secant Optimization Algorithm is a mathematically inspired approach derived from the classical secant method, offering a derivative-free and computationally efficient optimization strategy for high-dimensional problems^16^. In addition, improved variants of Particle Swarm Optimization (PSO) have been proposed by incorporating quadratic interpolation and local search mechanisms to enhance parameter estimation performance in solar photovoltaic systems^17^. Similarly, modified gradient-based algorithms that integrate quasi-Newton search rules with adaptive local search strategies have demonstrated improved efficiency in navigating high-dimensional optimization landscapes^18^. In addition, nature-inspired algorithms such as the moth-flame optimization method have been further enhanced through the introduction of local escape operators, enabling better avoidance of premature convergence and improved exploration of the solution space^19^.

These emerging methods demonstrate improved convergence behavior and solution accuracy in benchmark and engineering applications. Although they are not considered in the present study, their integration with machine learning models represents a promising direction for future hybrid forecasting frameworks. Despite these advancements, several limitations remain. First, many existing studies focus on a single predictive model or a single optimization strategy, without systematically evaluating the interaction between different model–optimizer combinations. Second, hyperparameter tuning is often performed using conventional or insufficient optimization techniques, which may lead to suboptimal performance in highly nonlinear and stochastic wind environments. Third, the relative effectiveness of emerging optimization algorithms, particularly in combination with advanced machine learning models, has not been comprehensively investigated. These gaps limit the ability to identify robust and generalizable forecasting frameworks suitable for real-world deployment.

Unlike prior studies that focus on a single hybrid configuration, this work systematically evaluates multiple model–optimizer combinations to reveal their interaction effects and identify robust forecasting strategies. To overcome these limitations, this study proposes a hybrid computational intelligence framework that integrates CatBoost (CAT), Long Short-Term Memory (LSTM), and Adaptive Neuro-Fuzzy Inference System (ANFIS) models with two nature-inspired optimization algorithms: Cuckoo Search Optimization (CSO) and the Stochastic Paint Optimizer (SPO). The framework is designed to systematically evaluate model–optimizer synergies and identify the most effective configuration for accurate wind power prediction under varying meteorological conditions. The main contributions of this study are as follows:

- Development of a unified hybrid forecasting framework for investigating interactions between machine learning, neuro-fuzzy, and metaheuristic optimization techniques in wind power prediction.
- Systematic comparative evaluation of CAT, ANFIS, CSO, and SPO combinations under identical experimental conditions to identify effective model–optimizer pairings.
- One of the first assessments of the Stochastic Paint Optimizer (SPO) for hyperparameter tuning in wind power forecasting applications.
- Comprehensive validation through feature importance analysis, multicollinearity assessment, cross-validation, and statistical significance testing.
- Practical insights into optimizer selection for renewable energy forecasting systems, with implications for forecasting reliability and grid operation.

## Materials and methods

### Dataset description

The dataset used in this study was obtained from a publicly available repository on Kaggle titled “Wind Power Generation Data Forecasting”. It consists of meteorological measurements and corresponding wind power output records collected from a wind energy system. The dataset contains approximately 43,800 samples, recorded from 1/2/2017 to 12/31/2021. The data are sampled at hourly frequency, enabling the capture of short-term variability in wind conditions and power generation. The dataset includes meteorological variables such as air temperature (2 m), relative humidity (2 m), dew point (2 m), wind speed at 10 m and 100 m, wind direction at both heights, and wind gusts at 10 m. The target variable represents the normalized power output of the wind generation system. It should be noted that the dataset does not explicitly specify the exact geographical location of the wind farm in the source. However, the data reflect realistic operational and meteorological conditions typical of utility-scale wind energy systems and are widely used for benchmarking forecasting models in the literature. Prior to model development, standard preprocessing steps were applied, including handling missing values, feature scaling, multicollinearity analysis, and transformation of directional variables into circular components. The dataset was then divided into training, validation, and testing subsets to ensure unbiased model evaluation.

**Fig. 1:**
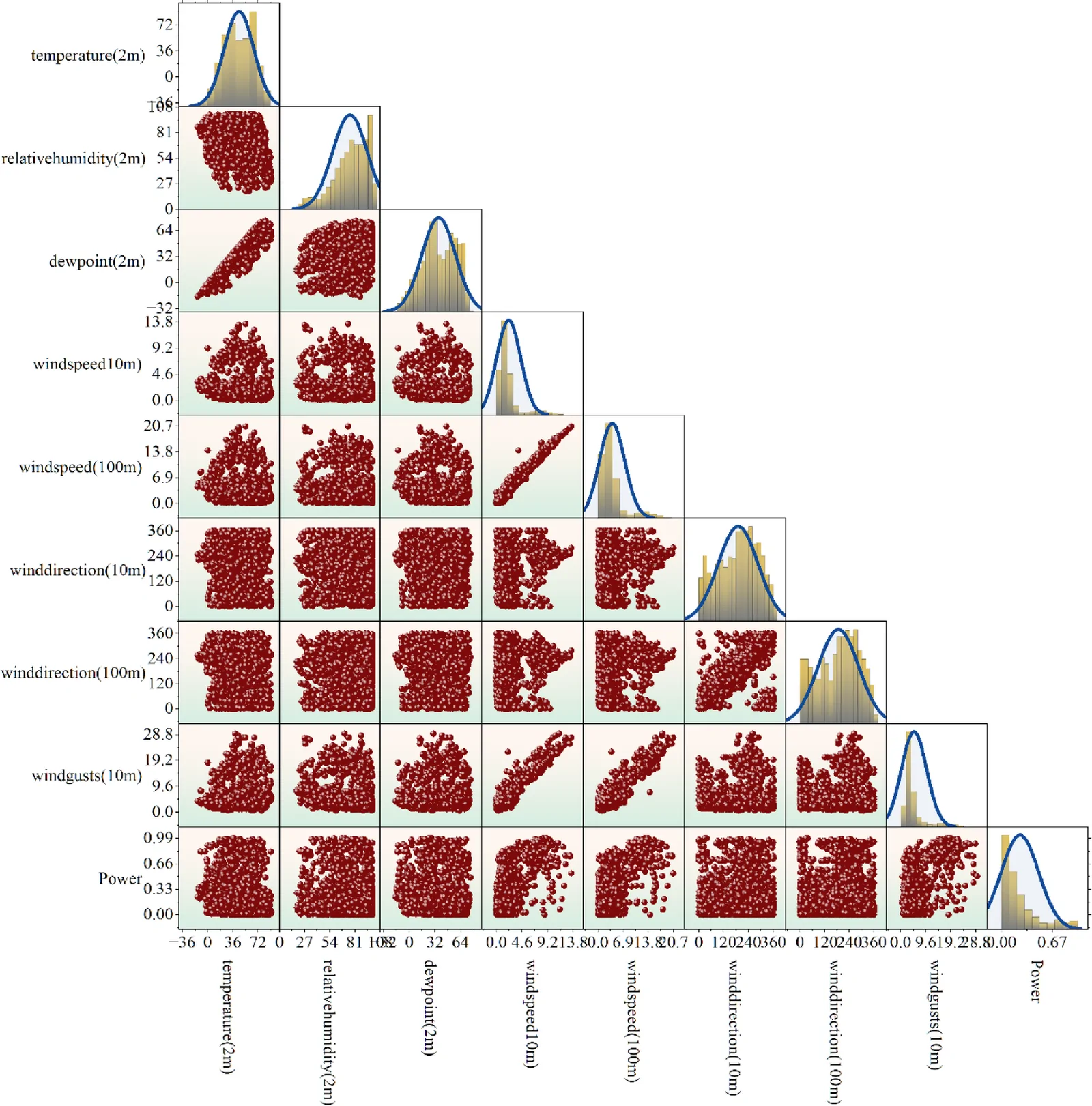
The relationships between input and output variables are assessed by utilizing a scatter matrix plot.

The dependent variable is the total electrical power generated by the installation, as recorded in the source data. For proper model building, the following standard preprocessing operations are strongly recommended: missing value imputation, detection and alleviation of multicollinearity (especially among wind speed variables), correct variable scaling, and conversion of wind direction to circular components (sine/cosine) to maintain angular relationships and improve model interpretability and performance. The scatter matrix plot in Fig. 1 provides a first glance at the correlations between the major meteorological factors that include the wind speed at different heights, the wind gusts, the temperature, and the relative humidity, and the output variable, that is, the wind power generation. Along the diagonal, histograms represent the distribution of the features. For example, these show that the wind speeds at the heights of 10 m and 100 m are heavily skewed towards the lower values, while the distribution of power reveals the range of the wind generation system in which it operates.

Table 1 presents an overview of the statistical properties of inputs and outputs utilized in the study. This particular height closely matches the hub height of wind turbines used in power stations, where the wind is usually stronger and less influenced by surface roughness. The data points for this variable ranged from 0.1 m/s to 20.65 m/s, with a mean of 3.886 m/s and a standard deviation of 3.474 m/s, indicating a wide range of wind conditions. Wind variation is very important for training predictive models, as it exposes them to scenarios of both low and high energy.

**Table 1:** Overview of input features and output variables with their statistical properties.

Variables	Data Role	Characteristics				
		Max	Min	Median	Average	St. Dev.
Temperature (2 m)	***Inputs***	90.600	−14.400	44.600	44.748	21.269
Relative humidity (2 m)		100.00	19.000	79.000	75.786	18.803
Dewpoint (2 m)		76.300	−17.100	35.400	36.588	21.021
Wind speed (10 m)		13.450	0.000	1.630	2.238	2.145
Wind speed (100 m)		20.650	0.100	2.970	3.886	3.474
Wind direction (10 m)		360.000	1.000	201.000	189.895	96.563
Wind direction (100 m)		360.000	0.000	199.000	186.543	99.036
Wind gusts (10 m)		29.200	0.600	3.400	5.095	4.825
Power	***Output***	0.991	0.000	0.150	0.241	0.248

**Fig. 2:**
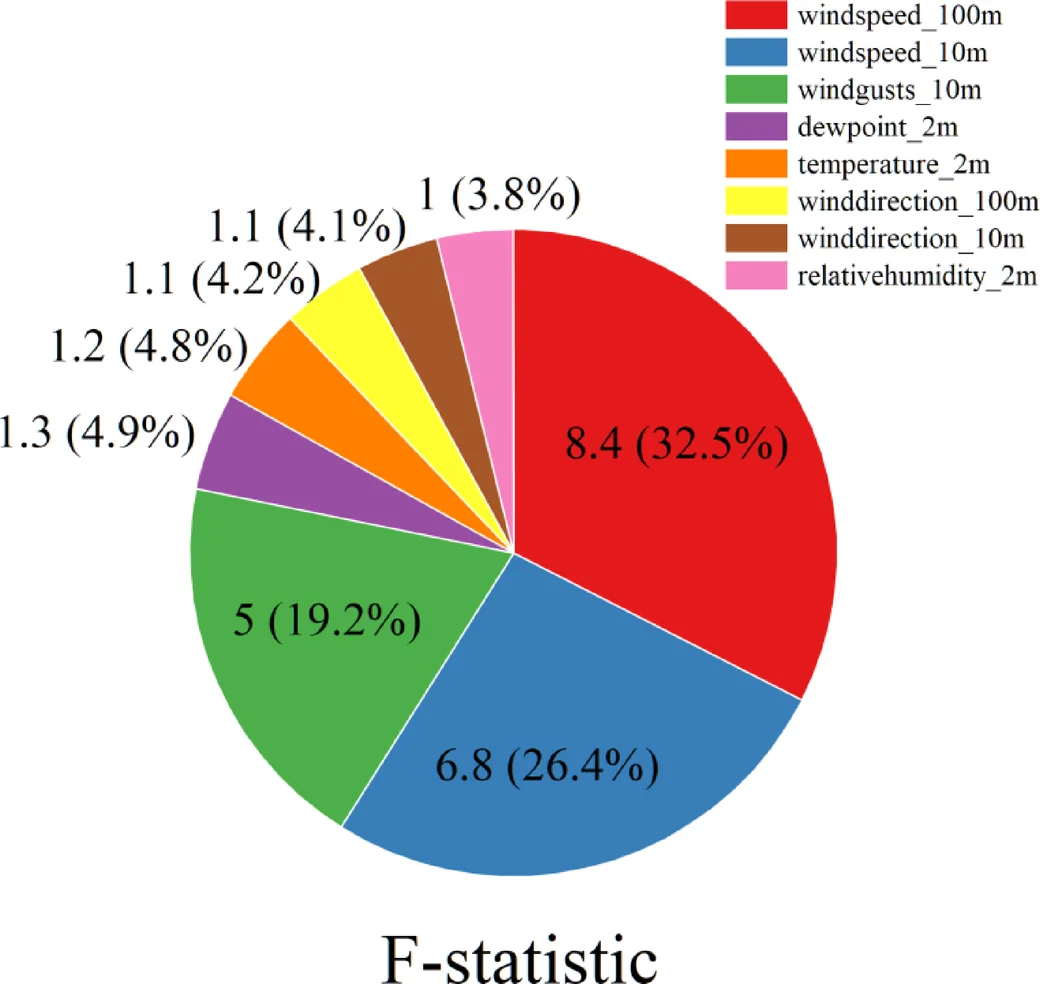
The F-statistic feature selection of the inputs and output variables.

The results of feature selection in Fig. 2 show that wind speed at 100 m is the most significant factor in determining wind energy output, accounting for 32.5% of the total importance, i.e., more than any other variable in the dataset. This discovery is exactly opposite to the way most of the modern utility-scale wind turbines have been set up, whose hub heights are usually at or above 100 m. At this height, the wind speed is normally higher and steadier; therefore, the energy captured from kinetic energy will be of higher value and more stable. In relation to Wind Power Generation, this is the most important factor that ensures the power remains efficient and reliable, as it highly depends on wind conditions at hub height. The feature importance ranking, where the wind speed at 100 m is at the top of the list, underscores its role as the nearest and, physically, most appropriate way to measure wind resources for the stability of wind turbines. Besides, small increases in wind speed at this height might result in a much larger increase in power generated, since the output is proportional to the cube of the velocity. Though the factors mentioned before, such as wind speed at 10 m and wind gusts, come forward as meaningful contributors to the feature importance, their total contribution is still less than that of wind speed at hub height. Meteorological variables like temperature, humidity, and wind direction are among those with only a slight influence, implying that only the most accurate, very high-resolution measurements of wind speed at 100 m are the most valuable inputs for forecasting models and decision-making processes in Wind Power Generation.

To ensure the robustness and generalizability of the models, a 5-fold cross-validation procedure was employed, as shown in Table 2. The dataset was partitioned into five subsets, where in each iteration, four subsets were used for training and one for validation. The average performance across all folds was used to assess model stability.

**Table 2:** Results of the 5-fold cross-validation.

Model	K1	K2	K3	K4	K5
***CAT***	0.9301	0.9275	0.9364	0.9328	*0.9398*
***LSTM***	0.930	0.9271	0.9359	0.9322	*0.9365*
***ANFIS***	0.9174	0.9261	0.9218	0.9283	*0.9307*

### CatBoost algorithm (CAT)

**Fig. 3:**
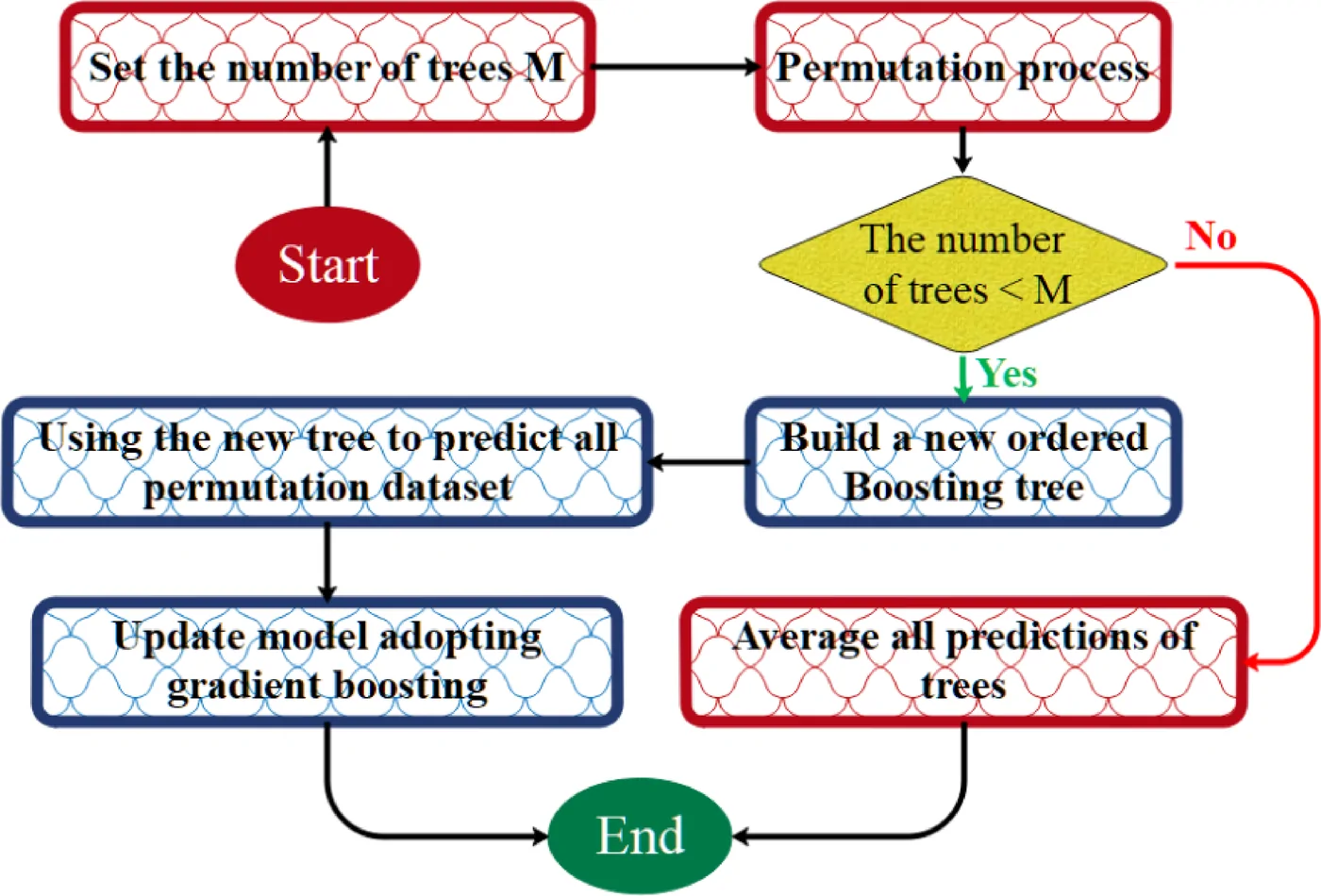
CAT boost flowchart.

For this research, CAT has been chosen to develop a model and forecast wind energy generation. Wind power is a complex, innovative system whose meteorological nature is nonlinear. Hence, Yandex’s gradient boosting decision tree algorithm, CatBoost, was designed to be the most efficient algorithm to handle a diverse dataset and achieve the highest prediction accuracy with minimal preprocessing; thus, its success in wind power generation. Furthermore, they include turbulent wind speed, direction, pressure, temperature, and turbine-specific parameters^20,21^. Most of the time, inputs in energy systems are exposed to variability and, more so, temporal changes, which put traditional predictive techniques in a hard spot. CatBoost addresses the issue by iteratively building a set of decision trees, where each tree is designed to nullify the remaining errors from previous trees. This sequential learning method empowers the model to uncover the wind power energy data’s complex patterns and nonlinear dependencies to a great extent. In addition, CatBoost can handle both numerical and categorical features simultaneously, so there is no need for one-hot encoding. Consequently, this is a good feature when dealing with different data types such as turbine ID, location, or modes of work. Furthermore, the use of ordered boosting and symmetric trees not only facilitates better model generalization but also reduces prediction bias, making it a strong candidate for wind energy forecasting, where overfitting and data imbalance problems have been identified. Moreover, by embodying CatBoost as a predictive tool for wind power generation, the study aims to enhance the accuracy of predicting crucial grid stability, energy market planning, and the overall incorporation of renewable energy sources into the national power infrastructure^22^. Figure 3 presents the flowchart of the CatBoost model.

### Adaptive neuro-fuzzy inference system (ANFIS)

**Fig. 4:**
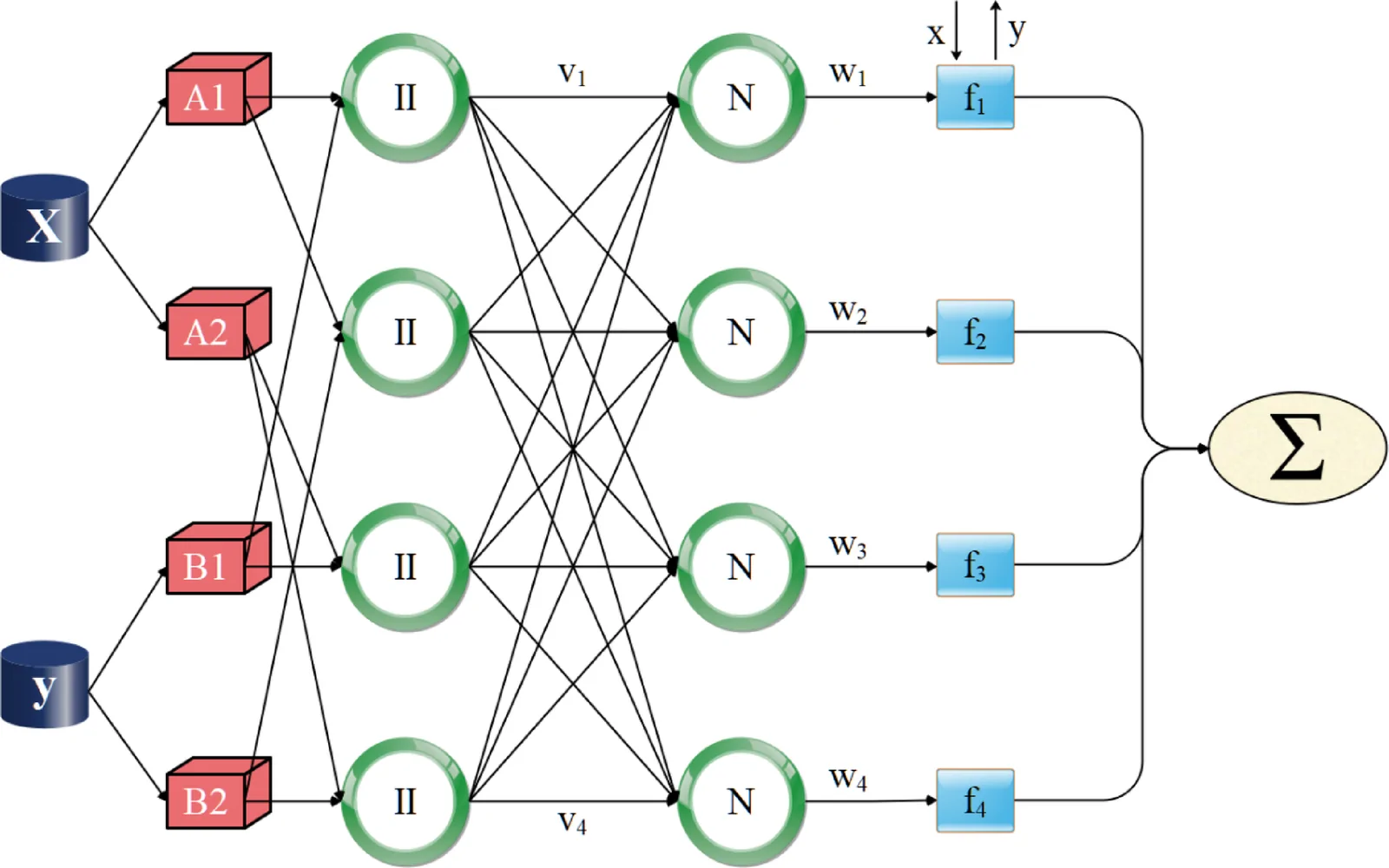
ANFIS Structure.

The ANFIS is an exceptional system that can model situations with many variables and uncertainties, such as wind-generated energy. Forecasting wind energy usually requires dealing with numerous variables that are difficult to predict, such as rapid changes in wind speed, fluctuations in atmospheric pressure, and slight changes in direction, which can vary even within an area or over a given period^23^. ANFIS delivers a model that is adept at learning the nonlinear relationships among these variables while also handling uncertainty and vagueness in the data, which, by the way, are key features of wind behavior. Essentially, ANFIS represents a blending of neural networks and fuzzy logic^24^. The neural element of the system enables it to extract the patterns from the historical data provided, e.g., previous wind measurements, and the resulting power outputs. Meanwhile, the construction of fuzzy logic services is a mode for dealing with indeterminate or linguistically defined quantities, e.g., ‘moderate wind’ or ‘high turbulence’, which are quite frequent in the context of meteorology. By interpreting these variables using fuzzy rules and continuously adjusting them through learning, ANFIS can make more accurate, adaptive predictions of wind-generated electricity under varying environmental conditions. In the context of wind power forecasting, ANFIS analyzes multiple inputs, such as wind speed, air density, temperature, and turbine-specific characteristics, to estimate future power outputs. This allows operators and planners to anticipate energy supply better, manage grid integration, and reduce dependency on fossil fuels. What makes ANFIS especially valuable in this domain is its ability to maintain interpretability: the rule-based structure not only delivers predictions but also reveals which combinations of environmental conditions most influence power generation. This can be useful for enhancing both technical planning and strategic decision-making in renewable energy systems. As wind power becomes a more integral part of the global energy mix, models like ANFIS help bridge the gap between the unpredictable nature of natural forces and the structured demands of the electricity infrastructure^25,26^. Its balance between learning efficiency and logical transparency makes it a practical and insightful tool for addressing the forecasting challenges unique to wind energy production. The flowchart of the ANFIS model is provided in Fig. 4.

### Long short-term memory (LSTM)

LSTM is a specialized type of recurrent neural network (RNN) designed to model sequential, time-dependent data by effectively capturing both short- and long-term temporal dependencies^27^. Unlike conventional RNNs, which often suffer from vanishing and exploding gradient problems during training, LSTM introduces memory cells and three gating mechanisms (input, forget, and output gates) that regulate the flow of information through the network. This architecture enables LSTM to selectively retain relevant historical information while discarding less useful data, making it particularly suitable for forecasting tasks involving complex temporal patterns. In wind power forecasting, LSTM has been extensively applied because wind generation is inherently influenced by continuously changing meteorological conditions, including wind speed, direction, temperature, and atmospheric pressure. By learning temporal relationships among these sequential observations, LSTM can capture nonlinear dynamics and seasonal fluctuations that are difficult to model with conventional statistical methods. Consequently, LSTM is often considered a strong baseline for renewable energy forecasting applications^28^.

### Cuckoo search algorithm (CSA)

The CSA is a metaheuristic that takes inspiration from nature and operates the same way as the breeding behavior of cuckoo birds does. In the wild, cuckoos tend to lay their eggs in the nests of other species and thus take advantage of the host birds, who will, without knowing, raise their young. This concept translates into the computational optimization world, where each “egg” represents a potential solution to a problem, and each “nest” denotes a location in the solution space^29^. When it comes to wind power generation, CSA is a solid plan to sort out hard, nonlinear problems like turbine placement, energy output forecasting, and load balancing within wind farms. The method is based on randomly distributing possible solutions over the search space, similar to cuckoos, which lay their eggs in different nests. Gradually, only the best solutions (those that closely meet the target, such as maximizing energy output or minimizing system loss) are kept and further developed, while the poor ones are discarded or replaced^30^. This operation reflects natural selection, and consequently, the algorithm will get closer to the optimum or a solution very close to it over time. One of the advantages of the CSA is that the algorithm can successfully combine local and global exploration, which means that the method can not only improve the already found good solutions (local search) but at the same time discover new parts in the solution space (global search) so as not to fall into one of the traps of premature convergence. The wind variables that are mentioned in nature - wind speed, direction, and turbine dynamics - are the reasons why the optimization landscape in wind energy systems is so complicated and fluctuating, which makes this CSA property very valuable. Implementing CSA will make it easier for evolved Ph.D. students in a laboratory setting to make decisions quickly and efficiently in the face of uncertainty. Systems will become more reliable, and the overall efficiency will increase. The algorithm’s adaptability and powerful search capabilities, which help address the challenges of accessing wind energy in the natural world, make it an effective tool^31^.

### Stochastic paint optimizer (SPO)

**Fig. 5:**
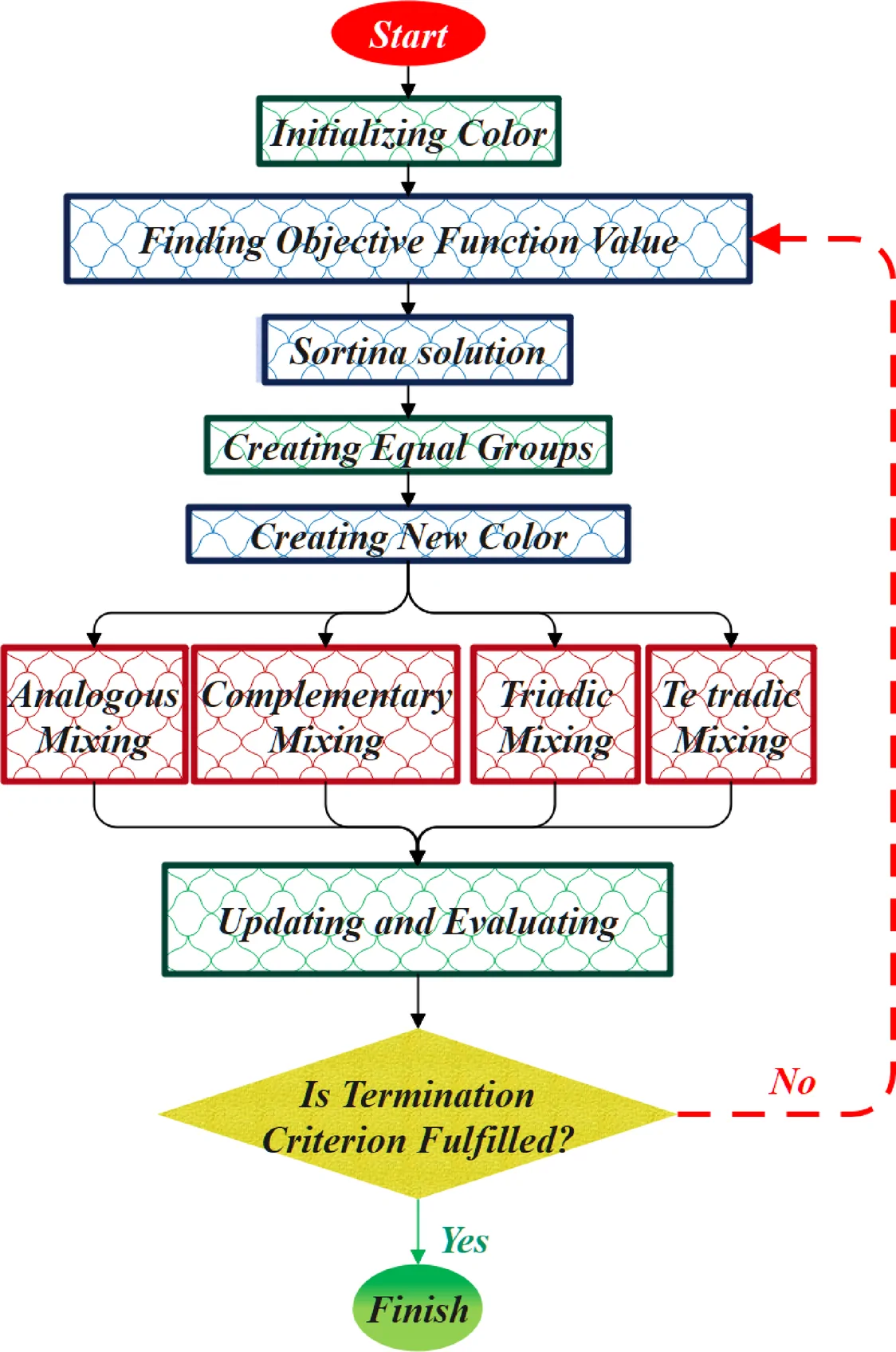
SPO flowchart.

The SPO is a novel optimization method rooted in nature and inspired by the process of creative painting, where different colors are combined and tested to get the most appropriate end product. In wind energy, SPO is a strong single-objective solution to the multi-conflicting problem of energy optimization, aiming to maximize output at minimal cost while not compromising the environment. The fact that SPO lacks internal control parameters is what sets it apart from others, making SPO usage more user-friendly and increasing its applicability to different kinds of problems. When the problem of energy optimization involves multiple conflicting objectives, the SPO is no longer a suitable solution, and the algorithm must be replaced by the Multi-Objective Stochastic Paint Optimizer (MOSPO)^32^. Thus, this one is a modified version of the former, equipped with an external archive and a dynamically selected leader. Both mechanisms help foster the variety of solutions available for the problem in question and, thus, will most likely reach the author of the trade-offs quite quickly. The issues related to the variability and uncertainty of wind energy systems that have to be mitigated by proper performance, reliability, and sustainability balance are precisely the ones where these characteristics can be of great help in solving^33,34^. Through MOSPO, the study of wind power generation can be conducted much more efficiently, as it can easily identify the optimal operating conditions and the most accurate methods for forecasting different scenarios. When there are complex system constraints, variations in environmental data, and the wind behaves in an apparently stochastic manner, the striking capability of MOSPO to model the wind system accurately makes it not only an excellent but also a very attractive solution. As a result of this operating approach, MOSPO can offer the wind energy sector multiple robust solutions for different performance metrics, thereby paving the way for the establishment of the most optimal and stable wind energy systems. Figure 5 presents the flowchart of the SPO optimizer.

### Performance evaluators

In wind power generation, various performance metrics are used to evaluate the accuracy and reliability of the proposed forecast models. Root Mean Square Error (RMSE) is the square root of the average squared error between the predicted and actual power; in other words, it shows the differences in the same units as the target variable is expressed (for example, megawatts). This metric has a high value in the wind energy area because it gives the greatest weight to the largest forecast errors, thereby reflecting the operational risks of abrupt overestimations or underestimations of wind power production. The Coefficient of Determination (R²) shows the proportion of the variance in actual values that is explained by the model. The R^2^ value close to 1 indicates that the model adequately describes the main weather factors that directly affect wind turbine power generation: temperature and pressure. The Scatter Index (SI) is an active measure of normalizing RMSE, which is close to the average power of the observed period. The index makes the relative size of the error visible and, as a result, helps compare the accuracy of different predictions of wind power at different times of day or at different locations, under different weather conditions or capacities. Mean Squared Error (MSE) is the average of all the squared deviations of the actual values from the predicted ones. Hence, large errors are severely punished in the method. Grid stability or market scheduling may be disrupted by large deviations in MSE. At the same time, Mean Absolute Error (MAE) measures the average absolute difference between actual and forecasted power and therefore serves as a simple indicator of the daily forecasting deviations that may be faced. The implementation of the mentioned measures, in this case, ensures the most complete forecasting performance evaluation, which takes error size and error frequency into account. Such a thorough approach is essential for developing models that can provide precise and reliable wind power predictions. Consequently, the system is stable, market operations are conducted efficiently, and large-scale integration of renewables is facilitated.

1 $$\:RMSE=\sqrt{\frac{1}{n}{\sum\:}_{i=1}^{n}{({y}_{i}-\widehat{{y}_{i}})}^{2}}$$

2 $$\:{R}^{2}=1-\frac{{\sum\:}_{i=1}^{n}{({y}_{i}-\widehat{{y}_{i}})}^{2}}{{\sum\:}_{i=1}^{n}{({y}_{i}-\stackrel{-}{y})}^{2}}$$

3 $$\:SI=\frac{RMSE}{\stackrel{-}{y}}$$

4 $$\:MSE=\frac{1}{n}{\sum\:}_{i=1}^{n}{({y}_{i}-\widehat{{y}_{i}})}^{2}$$

5 $$\:MAE=\frac{1}{n}{\sum\:}_{i=1}^{n}|{y}_{i}-\widehat{{y}_{i}}|$$

### Hybrid framework and optimization procedure

The proposed framework integrates machine learning models (CAT, LSTM, and ANFIS) with metaheuristic optimization algorithms (CSO and SPO) to enhance predictive performance through automated hyperparameter tuning. In this framework, each candidate solution generated by the optimizer represents a specific set of model hyperparameters. For the CAT model, the encoded parameters include tree depth and L2 regularization coefficient, while for the ANFIS model, the parameters include the number of fuzzy rules, batch size, and number of training epochs.

The optimization process begins with the random initialization of a population of candidate solutions. Each solution is evaluated by training the corresponding predictive model using the selected hyperparameters and computing the Root Mean Square Error (RMSE) on the validation dataset. During each iteration, the optimization algorithm updates the population by exploring the search space using its specific search strategy (e.g., Lévy flights in CSO or stochastic combination mechanisms in SPO). The objective is to minimize RMSE, thereby improving forecasting accuracy. The workflow of the proposed hybrid framework can be summarized as follows:

i. Initialize a population of candidate solutions (hyperparameter sets).
ii. Train the predictive model (CAT or ANFIS) using each candidate solution.
iii. Evaluate performance using RMSE.
iv. Update candidate solutions using the optimization algorithm.
v. Repeat until the stopping criterion is met (maximum iterations or convergence).
vi. Select the best-performing model configuration.

**Fig. 6:**
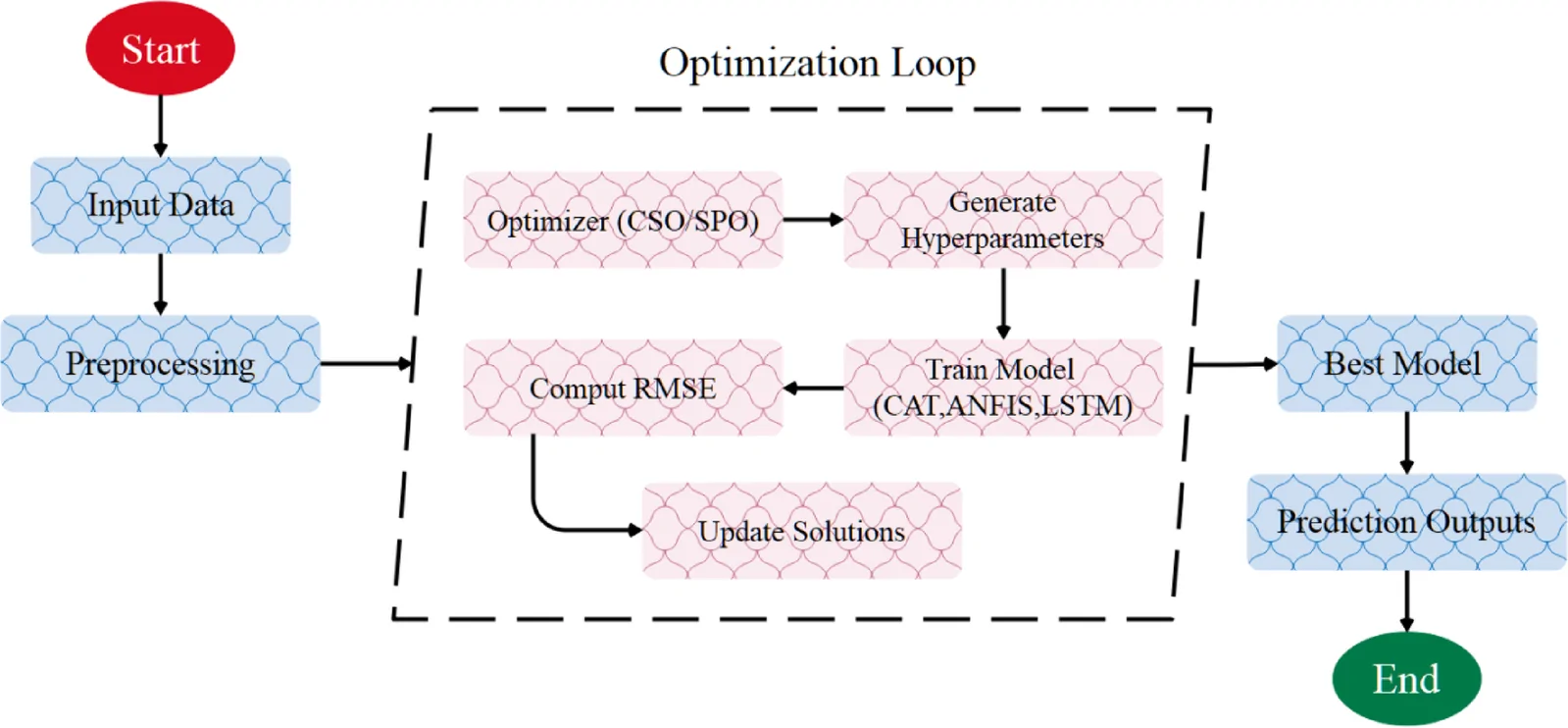
Proposed hybrid computational intelligence framework integrating predictive models (CAT, LSTM, and ANFIS) with metaheuristic optimization algorithms (CSO and SPO) for wind power forecasting.

This integration enables efficient exploration of the hyperparameter space and ensures that predictive models are optimally tuned to capture the nonlinear and stochastic characteristics of wind power generation. Figure 6 shows the proposed hybrid computational intelligence framework integrating predictive models (CAT and ANFIS) with metaheuristic optimization algorithms (CSO and SPO) for wind power forecasting.

## Results and discussion

### Models’ evaluation results

**Fig. 7:**
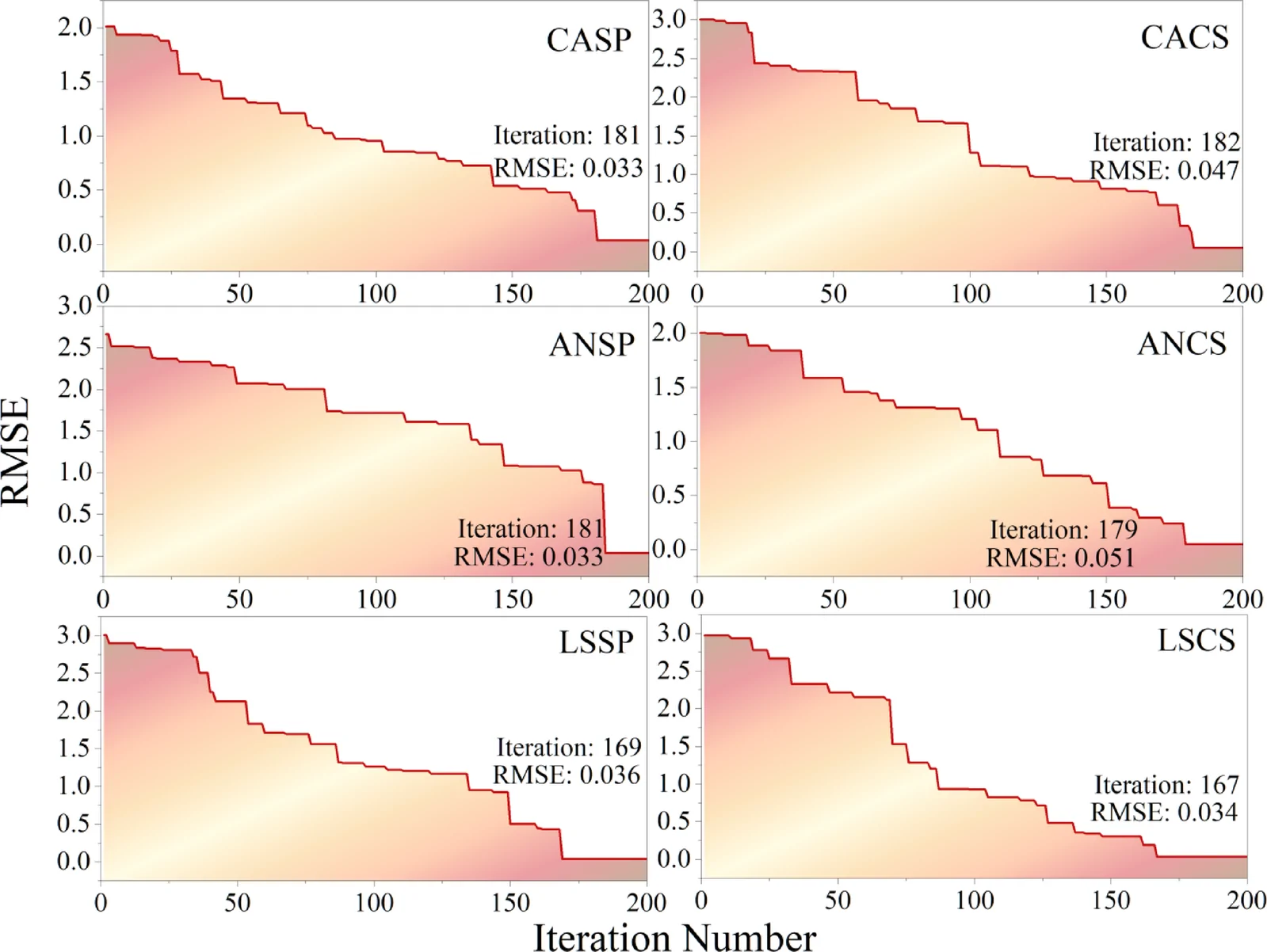
Convergence curves of the six hybrid forecasting models, illustrating the evolution of RMSE with optimization iterations.

All optimization algorithms were configured with a population size of 50 candidate solutions to ensure a balance between exploration and computational efficiency. The stopping criterion was defined as a maximum of 200 iterations or convergence based on the stabilization of the RMSE value, as illustrated in Fig. 7. For the CatBoost model, key hyperparameters including tree depth and L2 regularization coefficient (l2_leaf_reg) were optimized through the metaheuristic algorithms. For the ANFIS model, the structure was defined by the number of fuzzy rules (n_rules), while training was performed using mini-batch learning with specified batch sizes and epochs. For the LSTM model, the primary hyperparameters were the number of training epochs and the number of hidden units, selected to effectively learn temporal dependencies in sequential wind power data while maintaining computational efficiency. These parameters were similarly tuned using the optimization algorithms to achieve optimal predictive performance. The implementation details of the proposed framework can be made available as supplementary material to support reproducibility and future research.

Table 3 presents details of hybrid predictive models that show the parameters changed in various hyperparameter combinations, such as the number of metaheuristic-based hybrid iterations and neural network-driven hybrid epochs. Out of all the models, CASP has the highest number of iterations (149), which exceeded those of CACS (141) and CAT (119); thus, this longer iterative search implies that CASP searches deeper into the solution space to enable it to further adjust its parameters. One of the most important things about the optimization process is that it is specifically designed for wind power forecasting, which is quite difficult because the relationship between wind speed, wind properties, and power output is nonlinear and highly sensitive to small changes in input data.

CASP exploits the extra iterations to assimilate the slight influence of weather, select the most appropriate features, and tune the model’s parameters to reduce the prediction error. This, in turn, is very important for the situation of winds changing at different heights (for example, 10 m and 100 m), as even very small improvements in optimization can result in a more accurate power prediction. Moreover, such neural network–based hybrids as ANSP, ANCS, and ANFIS, just like other models, are trained by epochs, and among them, ANSP and ANFIS have 54 and 30 epochs, respectively, which is much less than CASP iterations, which means that they need less training time and they are converging faster, but less parameter tuning is done.

The LSTM model was trained for 50 epochs with 65 hidden units, providing sufficient capacity to capture temporal patterns in wind power generation without excessive model complexity. Compared with the hybrid models, LSTM relies on sequential feature learning rather than metaheuristic-guided parameter optimization, resulting in a simpler training process but fewer opportunities for global hyperparameter refinement. CASP, which has a large number of iterations, is therefore in a better position to deal with the random and changing nature of wind power. The comparison of hyperparameter configurations therefore highlights two complementary optimization strategies: iterative metaheuristic optimization for the CAT-based hybrid models and gradient-based temporal learning for LSTM. The superior forecasting performance of CASP indicates that the additional optimization provided by the metaheuristic search offers greater benefits than relying solely on deep sequential learning for this wind power forecasting application.

**Table 3:** The hyperparameters of the hybrid models, along with their assigned values.

Hyperparameter	Models								
	CASP	CACS	CAT	ANSP	ANCS	ANFIS	LSSP	LSCS	LSTM
Iterations	149	141	119	×	×	×	×	×	×
depth	8	7	4	×	×	×	×	×	×
l2_leaf_reg	7	6	3	×	×	×	×	×	×
Epochs	×	×	×	54	49	30	×	×	×
n_rules	×	×	×	13	11	4	×	×	×
batch_size	×	×	×	128	64	16	×	×	×
epochs	×	×	×	×	×	×	299	301	50
units	×	×	×	×	×	×	128	128	65

Table 4 offers a detailed comparison of the predictive performance of both single and hybrid models for training, validation, and testing datasets. In addition to the CAT and ANFIS models, a Long Short-Term Memory (LSTM) network was included as a deep learning benchmark due to its ability to capture temporal dependencies in sequential wind power data. The comparison is made in terms of key statistical indicators, such as RMSE, R², SI, MSE, and MAE. The results show the advantages of hybrid models over single models when modeling the complex, nonlinear nature of wind power generation. Among all models, CASP is the most accurate. In training, CASP achieves an R² of 0.9839, which is almost twice as good as that of CAT (0.9389), ANFIS (0.9296), and substantially higher than LSTM (0.9335), while at the same time obtaining the lowest RMSE (0.0337) and MAE (0.0274) values. The situation is essentially the same in validation and testing, with R² values of 0.9833 and 0.9841, respectively, and RMSE values not exceeding 0.035. These findings indicate that CASP is very efficient in capturing the intricate relationships between wind variables, such as wind speed and gusts, and turbine power output.

Furthermore, ANSP and CACS are also among the hybrid models that can beat the single ones in performance. However, they are not as good as CASP. For instance, ANSP can get the R² values close to 0.9810–0.9812, while CACS can go up to 0.9682–0.9691. Although LSTM achieved competitive performance among the single models, with R² values ranging from 0.9314 to 0.9349 and RMSE values between 0.0676 and 0.0694 across the three datasets, it consistently remained less accurate than the hybrid approaches. This suggests that while LSTM effectively captures the temporal characteristics of wind power generation, integrating machine learning models with metaheuristic optimization provides additional benefits by improving parameter optimization and nonlinear feature representation.

Single models like CAT, ANFIS, and LSTM, show much higher RMSE and MAE values than the leading hybrid models, which can be quite good, show much higher RMSE and MAE values, which indicate that they are less likely to be able to capture the stochastic variability that characterizes wind power generation. The main message that comes out of the above findings is the effectiveness of hybrid modeling, in particular CASP, in enhancing wind forecast precision. The high R² and low error metrics indicate that CASP can be a useful tool for facilitating operational planning, grid integration, and energy market strategies by providing accurate predictions of power output under different wind conditions. Overall, the comparison demonstrates that the proposed hybrid computational intelligence framework not only surpasses conventional machine learning models but also outperforms the deep learning-based LSTM benchmark, confirming its robustness and suitability for accurate wind power forecasting under variable operating conditions.

**Table 4:** Performance metrics of the models, assessing their predictive accuracy and effectiveness using key statistical indicators.

Process	Framework	Models	Evaluation Metrics				
			RMSE	R^2^	SI	MSE	MAE
***Train***	**Single**	*CAT*	0.0673	0.9389	0.1294	0.0045	0.0548
		*ANFIS*	0.0727	0.9296	0.1398	0.0053	0.0593
		*LSTM*	0.0680	0.9335	0.1308	0.0046	0.0305
	**Hybrid**	*CASP*	0.0337	0.9839	0.0648	0.0011	0.0274
		*CACS*	0.0472	0.9690	0.0908	0.0022	0.0385
		*ANSP*	0.0372	0.9805	0.0716	0.0014	0.0304
		*ANCS*	0.0570	0.9553	0.1097	0.0033	0.0467
		*LSSP*	0.0369	0.9804	0.0711	0.0014	0.0166
		*LSCS*	0.0341	0.9833	0.0657	0.0012	0.0154
***Validation***	**Single**	*CAT*	0.0673	0.9394	0.1306	0.0045	0.0551
		*ANFIS*	0.0740	0.9276	0.1436	0.0055	0.0606
		*LSTM*	0.0676	0.9349	0.1312	0.0046	0.0301
	**Hybrid**	*CASP*	0.0346	0.9833	0.0671	0.0012	0.0284
		*CACS*	0.0479	0.9682	0.0928	0.0023	0.0391
		*ANSP*	0.0368	0.9812	0.0715	0.0014	0.0300
		*ANCS*	0.0577	0.9548	0.1119	0.0033	0.0474
		*LSSP*	0.0360	0.9816	0.0698	0.0013	0.0160
		*LSCS*	0.0333	0.9843	0.0646	0.0011	0.0151
***Test***	**Single**	*CAT*	0.0684	0.9375	0.1317	0.0047	0.0557
		*ANFIS*	0.0739	0.9279	0.1423	0.0055	0.0607
		*LSTM*	0.0694	0.9314	0.1337	0.0048	0.0315
	**Hybrid**	*CASP*	0.0338	0.9841	0.0651	0.0011	0.0279
		*CACS*	0.0476	0.9691	0.0916	0.0023	0.0387
		*ANSP*	0.0574	0.9547	0.1105	0.0033	0.0472
		*ANCS*	0.0369	0.9810	0.0710	0.0014	0.0300
		*LSSP*	0.0374	0.9801	0.0720	0.0014	0.0171
		*LSCS*	0.0347	0.9829	0.0669	0.0012	0.0157

Figure 7 presents the convergence behavior of the six hybrid models by plotting RMSE against the iteration number. A lower RMSE indicates higher prediction accuracy, whereas a flattened convergence curve indicates that the optimization process has reached an optimal or near-optimal solution. Among all models, CASP exhibits the most stable convergence, reaching a final RMSE of approximately 0.033 after 181 iterations. The gradual reduction in RMSE demonstrates the effectiveness of the Stochastic Paint Optimizer in refining CatBoost hyperparameters and efficiently exploring the solution space. ANSP achieves a comparable final RMSE of approximately 0.033, though convergence is less smooth during intermediate iterations, indicating slightly lower optimization stability. Similarly, the LSTM-based hybrid models, LSSP and LSCS, show substantial improvements over the standalone LSTM model. LSCS converges to a final RMSE of approximately 0.034 after 167 iterations, while LSSP converges to approximately 0.036 after 169 iterations, demonstrating that integrating a metaheuristic optimization significantly enhances the predictive capability of LSTM networks. In contrast, CACS and ANCS converge to final RMSEs of approximately 0.047 and 0.051, respectively, indicating comparatively lower forecasting accuracy. Overall, the convergence profiles confirm that SPO-based optimization consistently produces faster, more stable convergence than CSO across both CatBoost- and LSTM-based frameworks. The superior convergence behavior of CASP, together with its lowest prediction error, highlights its suitability for reliable wind power forecasting, supporting improved turbine operation, maintenance scheduling, reserve allocation, and stable integration of renewable energy into modern power grids.

**Fig. 8:**
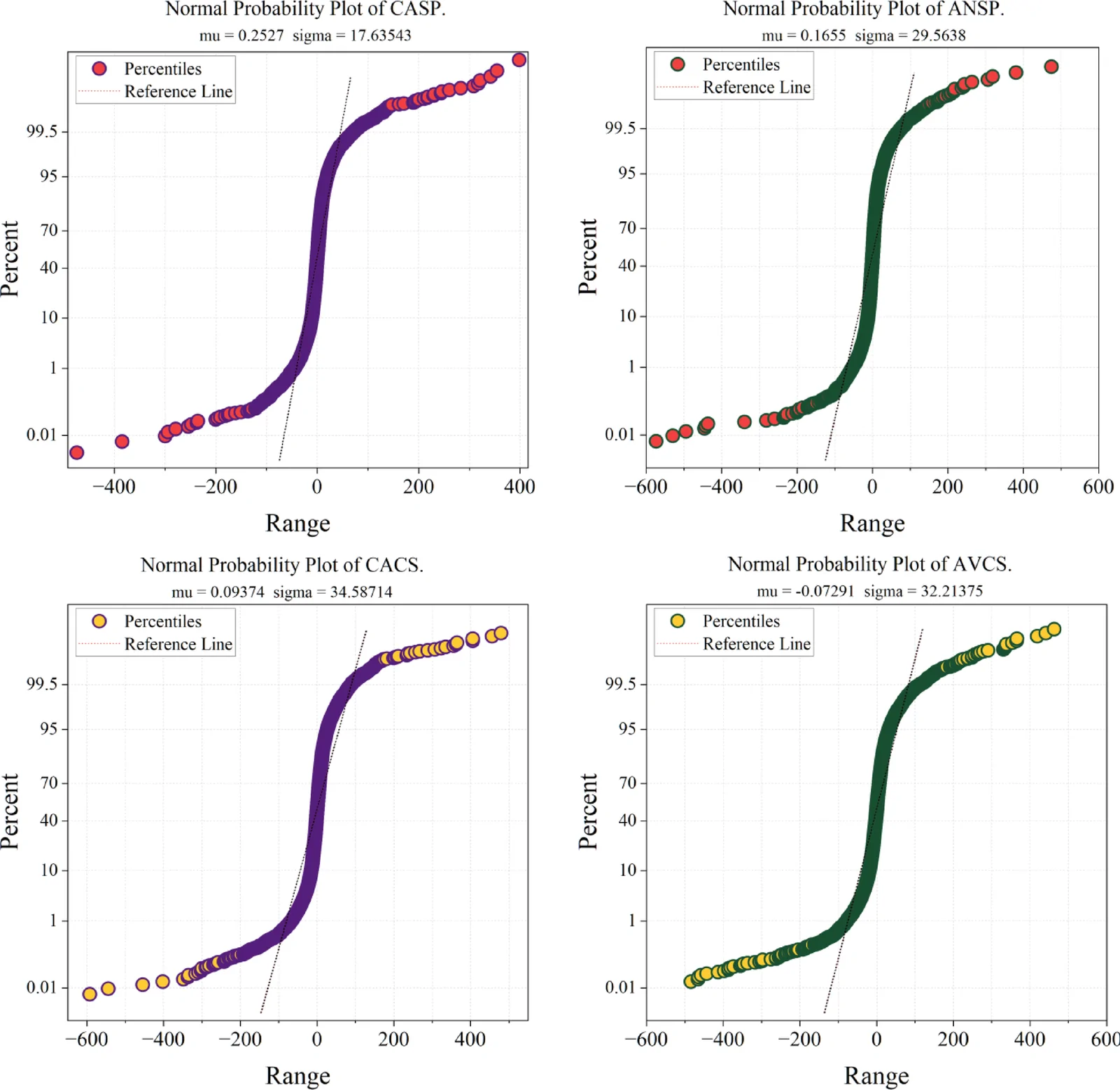

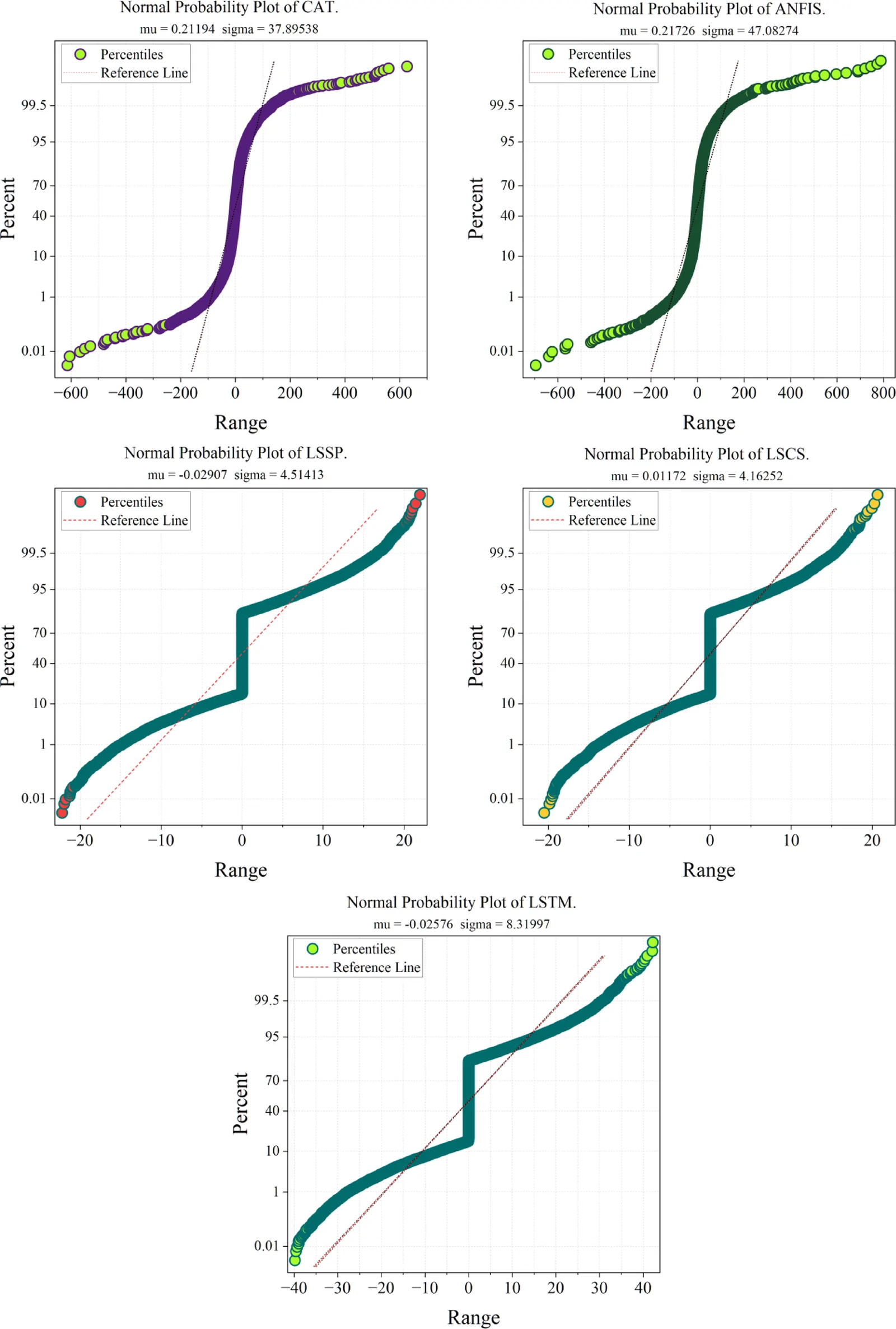
The probability plot errors of the proposed models.

Figure 8 depicts Normal Probability Plots (Q-Q plots) that assess the error distributions of the hybrid models, providing an indication of the accuracy and trustworthiness of the wind power predictions made by these models. Besides, the data points in these graphs, which are very close to the straight reference line, indicate that the model errors are approximately normally distributed, presuming that these are random rather than some systematic bias. In addition to the above, the plots present the average (µ) and the standard deviation (σ) of the errors, with the lowest values reflecting the errors that are very close to zero, from which it can be said that the model is desirable for consistent predictive performance. The CASP model, which represents the combination of the CAT model and SPO, is the one exhibiting the fewest errors. These errors almost trace the reference line, especially in the upper left region, where the mean is −0.2557, and the standard deviation is 17.6543. As a matter of fact, the CASP model gives out errors that are not only consistent but also normally distributed; therefore, it is the most reliable and the most precise option when it comes to forecasting the output of wind power. In comparison, other hybrid models, such as CACS (CAT combined with the CSO) and ANSP (ANFIS with SPO), exhibit broader error distributions and larger deviations at the tails, while the standalone CAT and ANFIS models show the widest error spreads, indicating the importance of optimization.

The probability plots of the LSTM-based models further support these findings. The standalone LSTM model exhibits a mean residual of −0.0258 and a standard deviation of 8.3200, indicating generally unbiased predictions but wider error dispersion than its optimized counterparts. Incorporating metaheuristic optimization substantially improves the distribution of residuals. LSSP achieves a mean residual of −0.0291 and a reduced standard deviation of 4.5141, while LSCS further decreases the standard deviation to 4.1625 with a mean residual of −0.0117, producing the narrowest residual distribution among the LSTM-based approaches. The closer alignment of the LSSP and LSCS residuals with the reference line indicates that optimization enhances the stability and consistency of LSTM predictions by reducing error variability while maintaining negligible prediction bias. Overall, the residual analyses confirm that optimization plays a critical role in improving forecasting reliability. While the optimized LSTM models significantly outperform the standalone LSTM by producing more concentrated and normally distributed residuals, CASP remains the most effective overall forecasting framework when considering the combined evidence from prediction accuracy, convergence behavior, and residual analysis. These findings demonstrate that integrating advanced learning algorithms with efficient metaheuristic optimization yields robust, reliable wind power forecasts suitable for practical grid integration and renewable energy management.

To evaluate the statistical significance of performance differences between models, the non-parametric Wilcoxon signed-rank test was employed. This test assesses whether the differences in prediction errors between two models are statistically significant without assuming normality of the data distribution. The results of the Wilcoxon test (Table 5) indicate that, although the CASP model achieves the lowest prediction error, the p-value (*p* = 0.1138) suggests that the improvement over some competing models is not statistically significant at the 0.05 significance level. However, for certain comparisons (e.g., ANSP), statistically significant differences are observed (*p* < 0.05).

The LSTM-based hybrid models (LSSP and LSCS) also performed competitively, with no statistically significant differences compared with CASP (*p* > 0.05). This indicates that incorporating metaheuristic optimization into LSTMs improves their predictive performance and enables them to achieve performance levels comparable to those of the proposed CASP framework. In particular, LSCS exhibited a smaller error gap than CASP, confirming the effectiveness of combining LSTM-based temporal feature extraction with Cuckoo Search Optimization. These findings indicate that while CASP demonstrates superior overall performance, the magnitude of improvement varies across models.

**Table 5:** Results of the statistical analyses based on the Wilcoxon test.

Model	Stat	*p*-value
***CASP***	182757231.5	0.1138
***CACS***	183,256,124	0.3446
***CAT***	183879698.5	0.6817
***ANSP***	180,402,677	0.0012
***ANCS***	184,293,602	0.8786
***ANFIS***	184131181.5	0.7968
***LSTM***	184008547.0	0.7245
***LSSP***	182941506.5	0.2147
***LSCS***	182615984.0	0.0876

**Fig. 9:**
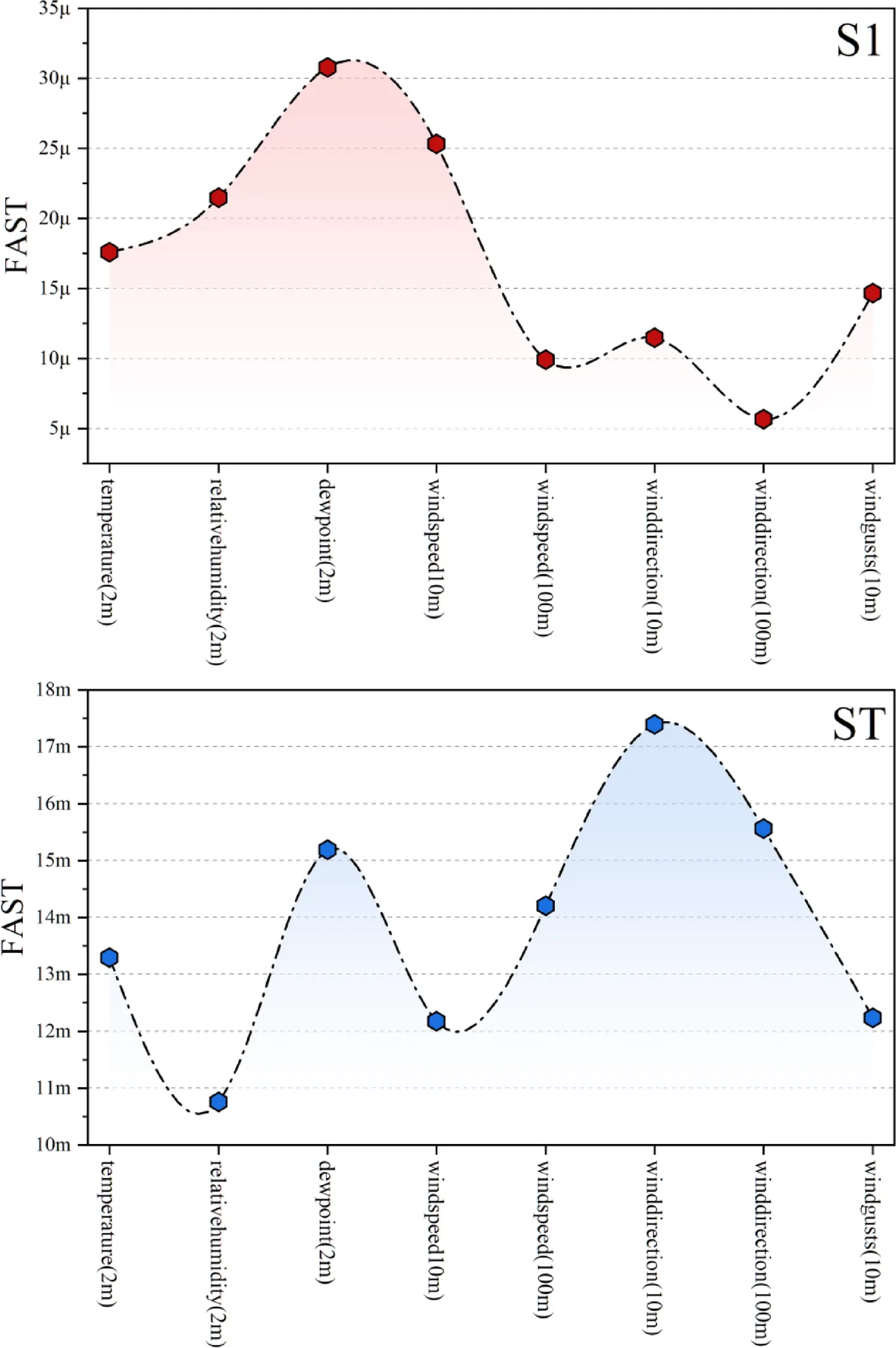
The FAST sensitivity analysis of the best-performing model.

The FAST sensitivity analysis is a diagrammatic exposition of the party-dependent meteorological features that affect the range of the best-performing hybrid model, i.e., the CASP model, which is illustrated by a wind power generation forecast (Fig. 9). The indices of both the main effects (S1) and overall effects (ST), which also take into account the interactions between different variables, are calculated. As a result, this analysis can provide a list of atmospheric factors that are the most obvious contributors to changes in turbine output, asserting a strong yet simple nature of their interaction. The main effect analysis (S1) shows that the dew point at 2 m is the largest single contributor to output variance, with Windspeed at 10 m being the next most important variable. The remaining variables, i.e., temperature, relative humidity, wind gusts, and wind direction, are only marginally responsible for the independent contributions. This fact, along with the physical mechanism by which wind speed is a key driver of wind power, indicates that the CASP model is distinctly sensitive to changes in dewpoint and lower-altitude wind speed when these inputs are handled individually. The variable-interaction-based total effect analysis (ST) alters the order of importance significantly. Firstly, we can find wind direction at 10 m as the main influential factor; secondly, despite the limited main effect, the two nearest ranges of wind direction at 100 m and dew point at 2 m also suggest themselves as the most significant contributors. The remarkable rise in the importance of wind direction compared to its main effect points to the presence of substantial interaction effects with other variables, especially wind speed and gusts. The FAST sensitivity experiment, in general, emphasizes the capabilities of the CASP model to reveal the complex dependencies of meteorological inputs on the production of wind energy. Although wind speed is essential for turbine output, the model’s elevated sensitivity to dew point and wind direction indicates its ability to capture nonlinear interactions that have a considerable impact on power generation.

Table 6 critiques the input features for the wind power generation study, highlighting multicollinearity and its impact on the best-performing hybrid model, CASP. When there is multicollinearity among input variables, the model’s stability may be compromised, and prediction accuracy may be reduced. Therefore, it is important to clean the feature set to ensure high accuracy of wind power forecasting. Although tree-based models such as CatBoost are generally robust to multicollinearity, the Variance Inflation Factor (VIF) was employed as a diagnostic measure to identify redundant features. Wind speed measurements at 10 m and 100 m exhibited strong collinearity, as they represent the same physical variable at different heights. From a domain perspective, wind speed at 100 m (hub height) has a more direct and physically meaningful relationship with wind turbine power output. Therefore, it was retained as the primary predictor. The 10 m wind speed variable was excluded to reduce redundancy and improve model interpretability. Preliminary analysis also indicated that including both variables did not significantly enhance predictive performance. Moreover, Windspeed (100 m) and wind gusts (10 m) also indicate high VIF scores of 10.70 and 11.20, respectively. At the same time, there are very low VIF scores for temperature (2 m), relative humidity (2 m), and wind direction at both altitudes, which give the minimum correlation and point to the independent contribution of these variables to the prediction of power output. Subsequent to considering multicollinearity, Windspeed (10 m) is eliminated from the feature set, whereas Windspeed (100 m) and wind gusts (10 m) continue on with significantly lower VIF scores (3.96 and 4.34, respectively). The preserved variables not only contain the essential characteristics of the wind but also contribute to predicting power more accurately, while the redundancy among variables has been minimized. This refinement enables the CASP hybrid model to efficiently integrate the most informative features without overfitting or instability. By zeroing in on Windspeed (100 m) and wind gusts (10 m) as the pivotal predictive inputs, the CASP model gets hold of the main factors that drive wind power generation. Besides, this is compatible with the physical principles that turbine output is very much affected by wind velocity and gust patterns. The careful selection of features and parameter reduction improve the model’s predictive accuracy, stability, and robustness, to the extent that the CASP’s excellence in forecasting wind power output is clearly demonstrated in this investigation.

**Table 6:** Description of features and determination of selected features.

NO.	Feature	VIF score-Initial feature vector	VIF score-Reduced feature vector
1	temperature_2m	1.130562	1.116531
2	relativehumidity_2m	1.400189	1.386582
3	windspeed_10m	26.116298	Remove
4	windspeed_100m	10.699535	3.956275
5	winddirection_10m	5.190836	5.189607
6	winddirection_100m	5.220231	5.218586
7	windgusts_10m	11.202409	4.337083

### Discussion

The CASP hybrid model that mixes CatBoost with the Stochastic Paint Optimizer is a model of trust beyond any doubt, as it can accurately predict the wind power generation 2 for which it was designed. Practically, a very low test RMSE of around 0.0338, an R² of about 0.984, and a very tight error distribution (σ ≈ 17.65) are the performance features that serve as confirmation of both the precision and the stability of the hybrid model under different wind conditions. In addition to CatBoost- and ANFIS-based models, LSTM and LSTM-based hybrid models (LSSP and LSCS) were evaluated to investigate the contribution of deep learning approaches in capturing temporal dependencies within wind power data. The standalone LSTM model achieved a test R² of 0.9314 and an RMSE of 0.0694, demonstrating its ability to learn sequential patterns but with lower accuracy than the proposed hybrid approaches. The improved performance of LSSP and LSCS relative to standalone LSTM highlights the effectiveness of metaheuristic optimization in improving neural network parameter selection. These models achieved test R² values of 0.9801 and 0.9829, respectively, showing that optimized LSTM architectures can effectively capture wind variability. However, CASP maintained superior performance, indicating that CatBoost is particularly suitable for structured wind forecasting datasets.

The superior performance of the CAT–SPO model compared to ANFIS-based hybrids can be attributed to differences in model structure and optimization efficiency. CatBoost, as a gradient boosting ensemble, is capable of capturing complex nonlinear interactions and feature dependencies in tabular data without requiring explicit rule definition. In contrast, ANFIS relies on a predefined fuzzy rule base, which may limit scalability and flexibility as the number of input variables increases. Furthermore, the optimization process appears to be more effective for CatBoost, as its hyperparameter space allows for smoother and more efficient exploration by the SPO algorithm. The combination of these factors contributes to the improved predictive accuracy observed for the CAT–SPO configuration.

Nevertheless, the quality and resolution of input data have a direct bearing on how confidently this model will perform, as do adequate computational resources that fuel its deep, iterative search, especially for real-time applications. The importance of this study is found in the very thorough comparison of hybrid configurations and the very clear demonstration of how optimizer-model synergies can materially improve wind power forecasting. Firstly, the research confirms that the SPO-tuned CatBoost is better than the one using only single learners and other mixed variants. Consequently, the meta-heuristic MIGHT can guide the hyperparameter to be more effective for the prediction of renewable energy. The employment of feature reduction through the Variance Inflation Factor (VIF), FAST sensitivity analysis, and several performance evaluation metrics differentiated by RMSE, R², SI, MAE, and MSE solidifies the methodological framework. Besides, the analysis also shows that while wind speed at the hub height appears to be the main driver, the second variables, such as dew point and wind direction, contribute significantly through interaction effects, which are by no means easily detected by univariate methods. Such discoveries more deeply delineate the influence of the atmosphere on wind power generation, and they are promising for scheduling operations, grid integration, and market solutions.

In fact, the next stages of the research might consider examining how CASP can be transferred by verifying it using data from wind farms located in different weather and terrain conditions. Moreover, incorporating the model with stochastic and ensemble-based forecasting, for example, via quantile regression, Bayesian techniques, or multi-optimizer hybrid ensembles, will certainly empower the provision of uncertainty quantification for operational risk management. Computational efficiency is a major development pathway; combining surrogate-assisted optimization, adaptive stopping criteria, or parallelized SPO execution cannot only drastically reduce the time required but also make the model more readily available for on-the-spot decision-making. Moreover, the deployment of such futuristic data streams like numerical weather prediction (NWP) outputs, lidar-based wind measurements, and micrositing variables may be a step ahead in improving forecast accuracy for medium- and long-term horizons. The transition of an AI model from a black box to a white box through explainable AI tools such as SHAP values and rule extraction will not only maintain users’ trust but also facilitate them in making prudent policy, operational, and investment decisions based on clear, physically meaningful predictive insights.

### Real-world implications of accurate wind power forecasting

There is only one feature of the forecasts that stands out above the rest, and that is their accuracy, which is a solid measure. Compared with standalone and LSTM-based approaches, the optimized CatBoost–SPO framework offers improved prediction reliability, enabling more precise estimation of future wind power generation. The measures of wind power that are delivered after tuning the hybrid CatBoost-ANFIS framework with CSO and SPO lead to a satisfying drop in the use of expensive spinning reserves through the shorter-term predictive accuracy. As a result, the frequency of departures is reduced, and the probability of lopping-off cases is lowered as well. Improved medium- and long-term forecasts can also lead to more active grid management, which can be achieved by reassessing maintenance planning and allocating reserves over long horizons. Moreover, market players will benefit significantly from an economic perspective. The owners of wind assets and traders can rework bidding strategies for the real-time and day-ahead markets to better match actual conditions, thereby increasing their revenues while lowering their risk. Utilities and power producers spend less on the system because forecast errors are lower, leading to lower imbalance penalty costs. Therefore, wind projects are becoming more economically efficient. The issue of operational asset management is also not left out, as it is pushed forward by reliable medium-term forecasts that form the basis of condition-based maintenance scheduling, which can prolong turbine service life and reduce the number of unplanned outages. Long-term production plans are better laid out if they include strategic capital decisions, such as repowering, capacity expansion, or site selection, which can be more easily integrated once alternative scenarios for expected energy production are clearer. One of the main advantages of enhanced forecasting is the increased ease of the technical integration of the renewable part of the energy system.

More accurate predictions allow the variable output of the wind road to be leveled, making the operation of transmission and distribution networks simpler. Hence, higher renewable penetration can be achieved without deteriorating the security of the system. By this smoothing effect, grid resilience is supported while still providing the decarbonization objectives. On the policy and investment side, improved forecasting also leads to a significant positive impact. Better forecasting sets the stage for ambitious carbon-neutral targets and appropriate market mechanisms. System operators who manage intermittency can become more efficient, thus getting closer to their ideal operating scenario. Investors experience the advantage of less doubt; hence, they can have deeper insight into project risks and potential return, which in turn can help pick up investments that are directed to the market. Moreover, there is a chain effect leading to environmental and social perks: through the limiting of renewable energy being taken over, that is, by fossil fuel backup generation, made possible by improved forecasts, the level of greenhouse gas emissions decreases; thus, air quality is improved. Communities, therefore, indirectly gain a certain level of stability in their electricity supplies and the opportunity that, as the system becomes more efficient, electricity prices may drop. Ultimately, one could say that the combined effect of these impacts is a strong example of how modern hybrid forecasting methods could be a leading force in advancing clean wind energy and supporting its transition to a low-carbon electricity system.

## Conclusions

This study examined wind power forecasting by comparing tree-based and neuro-fuzzy models tuned with nature-inspired optimizers. To address the challenge of accurate prediction, two metaheuristics, CSO and SPO, were applied to CAT and an ANFIS. The novelty lies in systematically pairing advanced learners with optimizers of contrasting characteristics: CS’s global search and SPO’s iterative refinement, yielding synergistic improvements across short-, medium-, and long-term horizons. Empirical results confirm the methodology’s effectiveness. The CAT + SPO hybrid (CASP) achieved superior performance, with a test RMSE of 0.0338 and R² ≈ 0.984, surpassing both standalone models and CS-tuned hybrids. Furthermore, the inclusion of LSTM and LSTM-based hybrid models (LSSP and LSCS) demonstrated that temporal learning mechanisms can effectively capture sequential wind variations; however, CASP maintained the highest predictive accuracy, confirming the suitability of optimized gradient boosting for structured wind power datasets. CASP’s optimization involved 149 configured iterations, showing stable convergence with RMSE plateauing after several hundred iterations—evidence of near-optimal parameter settings. Residual analysis further supported CASP’s robustness, with residuals closely matching a normal distribution (mean ≈ −0.26, σ ≈ 17.65), indicating low bias and stable predictions across wind conditions. Feature analysis highlighted hub-height wind speed (≈ 32.5%) as the dominant predictor, followed by wind gust at 10 m (≈ 19.2%), both with acceptable collinearity (VIF ≈ 3.96 and 4.34). FAST sensitivity analysis reinforced these findings: main effects emphasized moisture and lower-level wind speed, while total-effect indices revealed strong interactions involving wind direction at 10 m and 100 m. CASP effectively captured both primary drivers and nonlinear interactions (e.g., direction × speed, moisture effects) influencing turbine output. The practical implications are significant. More accurate short-term forecasts reduce reliance on costly spinning reserves and lower frequency deviations, minimizing load-shedding risk. Improved medium- and long-term forecasts enable better maintenance scheduling and reserve planning, enhancing economic performance by supporting refined bidding strategies, reducing imbalance penalties, and extending turbine lifespan through condition-based maintenance. For investors, more reliable yield projections facilitate repowering and expansion planning. At the system level, high-fidelity forecasts mitigate variability, enable greater renewable penetration, and strengthen grid security. Although LSTM-based hybrid models showed competitive performance, their reliance on sequential learning structures may require larger time-series datasets to fully exploit their capabilities. Therefore, the proposed CAT–SPO framework provides a more effective solution for the available wind power dataset by combining nonlinear feature learning and efficient parameter optimization. Among the evaluated forecasting models and hybrid configurations, CAT–SPO achieved the highest predictive accuracy and lowest error metrics. In such cases, the incorporation of temporal dependencies through lagged features or sequence-based modeling may be required. Future work will extend the proposed framework to multi-step and multi-horizon forecasting scenarios to further evaluate its applicability in real-world energy systems. Although the proposed CAT–SPO framework achieved the best performance among the evaluated models, the experimental comparison was limited to machine learning, neuro-fuzzy, and metaheuristic-based hybrid approaches. In addition, the proposed framework demonstrated consistent performance on a publicly available benchmark wind power dataset, its evaluation was limited to a single data source. The selected dataset was chosen because it provides realistic meteorological and wind power measurements and enables a controlled comparison among all forecasting models under identical experimental conditions. Nevertheless, wind characteristics vary considerably across geographical regions, turbine types, and operating environments. Consequently, the generalizability of the proposed framework should be further investigated using multiple benchmark datasets representing different climatic conditions, wind farm locations, and forecasting horizons. This constitutes an important direction for future research.

## Data Availability

Dataset will provide under reasonable request.

## Code availability

The source code used to develop and evaluate the proposed framework is publicly available on GitHub at https://github.com/Tariqalkhrissat10/Wind-Power-Forecasting-and-Grid-Integration-Applications.

## References

[1] Dorostkar, E. Quantum computing-artificial intelligence synergy for adaptive urban morphogenesis: Modeling China’s hyper-growth cities under uncertainty. *J Chin. Archit. Urban*, **8** (2), 25250046 (2025).

[2] He, Y. C. et al. Short-term prediction of wind vector at multi-heights via deep learning techniques based on marine measurements from Light-Detection-and-Ranging device. *Phys Fluids*, **38**(1), 015111 (2026).

[3] Hou, W., Hou, L., Zhao, S. & Liu, W. A hybrid data-driven robust optimization approach for unit commitment considering volatile wind power. *Electr. Power Syst. Res.* **205**, 107758 (2022).

[4] Yang, M. & Guo, Y. Error correction method of ultra-short-term prediction based on load peak-valley characteristics for wind farm cluster. *CSEE J. Power Energy Syst.* **12** (1), 258–270 (2026).

[5] Xie, Q., Zheng, Z., Guo, Y. & Xiao, X. Voltage Stability and Support in Wind Farm Integrated LCC-HVDC Systems During FRT. *IEEE Trans. Power Syst*, **41** (1), 731–746 (2025).

[6] Su, L. et al. An advanced energy-coordinated control for half-bridge submodule-based centralized DC chopper in offshore wind MMC-HVDC systems. *IEEE Trans. Power Electron*, **41** (7), 11765–11779 (2026).

[7] Wang, M., Cheng, F., Xie, M., Qiu, G. & Zhang, J. Intensive multiorder feature extraction for incipient fault detection of inverter system. *IEEE Trans. Power Electron.* **40** (2), 3543–3552 (2024).

[8] Yang, Y., Yan, G., Mu, G. & Chen, Z. Hierarchical bidding strategy for heterogeneous P2H loads and wind power: state-driven aggregation and switching time scheduling. *Energy*, **334**, 137766, (2025).

[9] Lin, P. et al. Power lever: To transform interlinking architecture in hybrid ac/dc microgrids community. *IEEE Trans. Ind. Electron*, **73** (5), 6984–6997 (2025).

[10] Agbakwuru, V., Obidi, P. O., Salihu, O. S. & MaryJane, O. C. The role of renewable energy in achieving sustainable development goals. *Int. J. Eng. Res. Updat*. **7**, 13–27 (2024).

[11] Ma, L. *IEEE 5th International Electrical and Energy* Conference *(CIEEC)*, IEEE, 2022, pp. 2615–2620. (2022).

[12] Wadirin, W. et al. Comprehensive overview of wind turbines. *Indones J. Eng. Sci.* **5**(3), 13–18 (2024).

[13] Khan, M. A. Advancing Sustainable Power: A Comprehensive Review of Technologies and Strategies for Green Electricity Production, *Collect J Chem Eng.* ; 1 ART0039, p. 2, 2024. (2024).

[14] Pawar, S. Harnessing the power of renewable energy: A study of sustainable sources and technologies. *Res. J. Appl. Sci.* **3**, 163–169 (2024).

[15] Hussein, N. K. et al. Schrödinger optimizer: A quantum duality-driven metaheuristic for stochastic optimization and engineering challenges. *Knowledge-Based Syst*, **328**, 114273, (2025).

[16] Ibrahim, M. Q., Qaraad, M., Hussein, N. K., Farag, M. A. & Guinovart, D. Secant Optimization Algorithm for efficient global optimization. *Sci Rep*, **16**, 6659 (2026). 10.1038/s41598-026-36691-z PMC12914007 41617807

[17] Qaraad, M. et al. Quadratic interpolation and a new local search approach to improve particle swarm optimization: Solar photovoltaic parameter estimation. *Expert Syst. Appl.* **236**, 121417 (2024).

[18] Mahmood, B. S., Hussein, N. K., Aljohani, M. & Qaraad, M. A modified gradient search rule based on the quasi-newton method and a new local search technique to improve the gradient-based algorithm: Solar photovoltaic parameter extraction. *Mathematics* **11** (19), 4200 (2023).

[19] Qaraad, M. et al. Photovoltaic parameter estimation using improved moth flame algorithms with local escape operators. *Comput. Electr. Eng.* **106**, 108603 (2023).

[20] Geeitha, S., Ravishankar, K., Cho, J. & Easwaramoorthy, S. V. Integrating cat boost algorithm with triangulating feature importance to predict survival outcome in recurrent cervical cancer. *Sci. Rep.* **14** (1), 19828 (2024). 39191808 10.1038/s41598-024-67562-0 PMC11349876

[21] Xiao, X. et al. Integrated Decision-Making Methodology for Low-Carbon Data Center Siting, Dispatch, and Transmission Based on Wind-Solar-Hydro-Computing Synergy. *IEEE Trans. Ind. Appl*, 1–11 (2026).

[22] Tian, G. et al. A Dual-Engine Artificial Intelligence Framework Accelerates Sustainable Aviation Fuel Component Synthesis. *J Am. Chem. Soc*, **148** (9), 9879–9891 (2026). 10.1021/jacs.5c22256 41725375

[23] Deif, M., Hammam, R. & Solyman, A. Adaptive neuro-fuzzy inference system (ANFIS) for rapid diagnosis of COVID-19 cases based on routine blood tests, International *Journal of Intelligent Engineering and Systems*, **14**, 178–189 (2021).

[24] Zhang, C. et al. A multiple model type-3 fuzzy control for offshore wind turbines using the Active Rotary Inertia Driver (ARID). *Ocean. Eng.* **313**, 119337. 10.1016/j.oceaneng.2024.119337 (2024).

[25] Thandra, J. & Basha, S. S. Artificial intelligence (AI) model: Adaptive neuro-fuzzy inference system (ANFIS) for diagnosis of COVID-19 influenza. *Comput. Inf.* **41** (4), 1114–1135 (2022).

[26] Jiang, Y., Wang, J., Zhao, H. & Terzija, V. Dynamic Interval State Estimation for Integrated Electricity and Heating Systems. *IEEE Trans. Power Syst*, **41** (1), 82–95 (2025).

[27] Wan, A., Du, C., AL-Bukhaiti, K. & Chen, P. Optimizing combined-cycle power plant operations using an LSTM-attention hybrid model for load forecasting. *J. Mech. Sci. Technol.* **39** (10), 6371–6380. 10.1007/s12206-025-0961-3 (2025).

[28] Al-azazi, F. A. & Ghurab, M. ANN-LSTM: A deep learning model for early student performance prediction in MOOC. *Heliyon* **9** (4), e15382. (2023). 10.1016/j.heliyon.2023.e15382 37089306 10.1016/j.heliyon.2023.e15382 PMC10119763

[29] Al-Abaji, M. A. A literature review of cuckoo search algorithm. *J. Educ. Pract.* **11** (8), 1–8 (2020).

[30] Khrissi, L., El Akkad, N., Satori, H. & Satori, K. Simple and efficient clustering approach based on cuckoo search algorithm, in *Fourth International Conference On Intelligent Computing in Data Sciences (ICDS)*, IEEE, pp. 1–6., (2020).

[31] Shehab, M. & Khader, A. T. Modified cuckoo search algorithm using a new selection scheme for unconstrained optimization problems. *Curr. Med. imaging*. **16** (4), 307–315 (2020). 32410534 10.2174/1573405614666180905111128

[32] Kaveh, A., Talatahari, S. & Khodadadi, N. Stochastic paint optimizer: theory and application in civil engineering. *Eng Comput*, **38**, 1–32 (2020).

[33] Khodadadi, N., Mirjalili, S. M., Mirjalili, S. Z. & Mirjalili, S. Chaotic stochastic paint optimizer (CSPO), in *Proceedings of 7th International Conference on Harmony Search, Soft Computing and Applications: ICHSA 2022*, Springer, 195–205. (2022).

[34] Khodadadi, N., Abualigah, L. & Mirjalili, S. Multi-objective stochastic paint optimizer (MOSPO). *Neural Comput. Appl.* **34** (20), 18035–18058 (2022).

